# Highly Efficient Composite Flame Retardants for Improving the Flame Retardancy, Thermal Stability, Smoke Suppression, and Mechanical Properties of EVA

**DOI:** 10.3390/ma13051251

**Published:** 2020-03-10

**Authors:** Yilin Liu, Bin Li, Miaojun Xu, Lili Wang

**Affiliations:** College of Chemistry, Chemical Engineering and Resource Utilization, Northeast Forestry University, Harbin 150040, China; Liuyilin_1111@163.com (Y.L.); libinzh62@163.com (B.L.); miaojunxu@163.com (M.X.)

**Keywords:** polymer, rare earth elements, composite flame retardant, flame retardancy, mechanical properties

## Abstract

Ethylene vinyl acetate (EVA) copolymer has been used extensively in many fields. However, EVA is flammable and releases CO gas during burning. In this work, a composite flame retardant with ammonium polyphosphate (APP), a charring–foaming agent (CFA), and a layered double hydroxide (LDH) containing rare-earth elements (REEs) was obtained and used to improve the flame retardancy, thermal stability, and smoke suppression for an EVA matrix. The thermal analysis showed that the maximum thermal degradation temperature of all composites increased by more than 37 °C compared with that of pure EVA. S-LaMgAl/APP/CFA/EVA, S-CeMgAl/APP/CFA/EVA, and S-NdMgAl/APP/CFA/EVA could achieve self-extinguishing behavior according to the UL-94 tests (V-0 rating). The peak heat release rate (pk-HRR) indicated that all LDHs containing REEs obviously reduced the fire strength in comparison with S-MgAl. In particular, pk-HRR of S-LaMgAl/APP/CFA/EVA, S-CeMgAl/APP/CFA/EVA and S-NdMgAl/APP/CFA/EVA were all decreased by more than 82% in comparison with pure EVA. Furthermore, the total heat release (THR), smoke production rate (SPR), and production rate of CO (COP) also decreased significantly. The average mass loss rate (AMLR) confirmed that the flame retardant exerted an effect in the condensed phase of the composites. Meanwhile, the combination of APP, CFA, and LDH containing REEs allowed the EVA matrix to maintain good mechanical properties.

## 1. Introduction

Ethylene vinyl acetate (EVA) copolymer is used extensively in many different fields, such as the wire, cable, and wrapper industries [1]. However, EVA is flammable and releases CO gas during burning [2]. In recent years, layered double hydroxides (LDHs) and intumescent flame retardants (IFRs) have been used to increase the flame retardancy and smoke suppression of the EVA matrix [3,4]. The LDH is composed of positively charged brucite-like layers, meanwhile containing charge-compensating anions and water molecules in the interlayer gallery [5]. The general formula of LDH is [M^2+^_1−x_M^3+^_x_(OH)_2_]^x+^[A^n−^]_x/n_·mH_2_O [6], where M^2+^ and M^3+^ are divalent and trivalent cations that occupy octahedral positions in the hydroxide layers. A^n−^ denotes the anions in the interlayer. X stands for the cationic ratio of M^3+^/(M^2+^ + M^3+^) and it can be adjusted [7]. LDH can be used in flame retardation extensively due to its advantages, such as low smoke, non-toxicity, and no harmful gas generation [8]. During burning, LDH releases water vapor and carbon dioxide that can cool the species and dilute the surrounding flammable gas to prevent the burning of composites [9].

**Figure 1:**
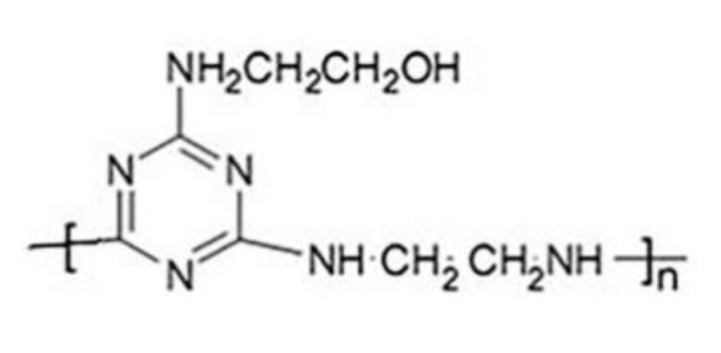
Structure of the charring–foaming agent (CFA).

IFRs has no halogen atoms, exhibits low toxicity, and has a small amount of smoke release [10]. Meanwhile, IFRs can generate a thermally stable char layer during burning, which can protect the underlying material from fire. A charring–foaming agent (CFA) plays the role of charring agent. During combustion, the acid source’s reaction can promote the dehydration and carbonization of the charring agent to form a char layer [11]. The structure of CFA is shown in Figure 1. The reported IFRs consisted of ammonium polyphosphate (APP) and CFA; when the mass ratio of APP and CFA was equal to 3:1, the EVA matrix had the best flame retardancy [12]. Moreover, composite flame retardants including APP, CFA, and LDH containing a transition element have been used to enhance the flame retardancy of EVA [13].

Rare-earth elements are an important material that can be used in many fields such as global communications, medicine, agriculture, batteries, and hybrid engines due to their electronic, magnetic, and catalytic properties [14,15,16,17,18]. However, the influence of a composite flame retardant including REEs, LDHs, and IFRs on the flame retardancy of EVA has not been reported. In this paper, APP and CFA with a mass ratio of 3:1 were combined with LDH doped with trace amounts of REEs (La, Ce, Nd) to obtain a novel composite flame retardant of EVA. The new flame-retardant system not only maintained their mechanical properties well, but also achieved excellent flame retardancy and smoke suppression.

## 2. Materials and Methods

### 2.1. Materials

Ammonium polyphosphate (99%) was obtained from Samsung Company, Seoul, Korea. The charring–foaming agent was synthesized in our laboratory. Sodium hydroxide (99%), sodium stearate (99%), magnesium nitrate (99%), aluminum nitrate (99%), and sodium carbonate (99%) were purchased from Tianjin Fuchen Chemical Reagent Factory, Tianjin, China. Lanthanum nitrate, cerium nitrate, and neodymium nitrate were obtained from Tianjin Guangfu Fine Chemical Research Institute, Tianjin, China. EVA was supplied by Arkema Company, Paris, France. All chemicals were analytical grade without further purification.

### 2.2. Methods

#### 2.2.1. Synthesis of the Modified LDHs

A mixed aqueous solution of Mg(NO_3_)_2_ and Al(NO_3_)_3_ with Mg^2+^ and Al^3+^ in a molar rate of 4:1 was adjusted to a constant pH 8–9 under stirring at 70 °C. Then, a 0.25 g amount of sodium stearate was mixed with the above solution and reacted for 2 h. The prepared precursor was put in a microwave oven at 70 °C for 30 min. The precipitate was washed with deionized water to pH = 7, then filtered and dried. The sample was called S-MgAl. The LDH containing REEs (La, Ce, Nd) was obtained by the same method with a REEs^3+^:Mg^2+^:Al^3+^ molar rate of 0.05:4:0.95. The samples were called S-LaMgAl, S-CeMgAl, and S-NdMgAl, respectively.

#### 2.2.2. Preparation of CFA

**Figure 2:**
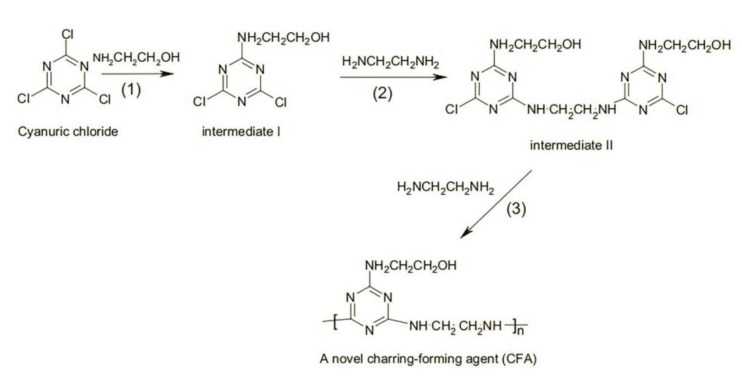
Scheme of the synthetic CFA.

The synthetic scheme of CFA is shown in Figure 2 [19]. The experimental device consisted of a 1000 mL four-neck flask, a stirrer, a thermometer, a reflux condenser, and a dropping funnel. First, 0.4 mol cyanuric chloride and 200 mL acetone were added into the four-neck flask. A solution of ethanolamine in acetone and a solution of sodium hydroxide were simultaneously added dropwise into the four-neck flask and the experimental temperature was kept at 0–5 °C. The obtained reactive system was called intermediate I.

A mixed aqueous solution containing 0.5 mol ethylenediamine and 1.0 mol sodium hydroxide was added into intermediate I. The reaction temperature was kept at 40–50 °C for over 10 h. After cooling, the product was filtered, washed, and dried to obtain intermediate II (yield: 99.4%).

A 0.5 mol amount of intermediate II and a mixed solution containing 0.5 mol ethylenediamine and 1.0 mol sodium hydroxide in 500 mL water were added into a 2000 mL four-neck flask provided with a stirrer, a thermometer, and a reflux condenser, and the reacting solution was kept under reflux for 10 h. Then, the obtained solution was filtered, washed, and dried to obtain the charring–foaming agent [20]. The structure of CFA has already been demonstrated [21].

#### 2.2.3. Preparation of Composites

The mass of the flame retardant accounted for 20%. The composites’ ingredients are listed in Table 1. The composites were prepared via the melt-blending method at 140 °C in an RM-200A torque rheometer (Harbin University of Science and Technology, Harbin, China) for 6 min with a rotor speed of 60 rpm. The composites were called S-MgAl/EVA, S-LaMgAl/EVA, S-CeMgAl/EVA, S-NdMgAl/EVA, APP/CFA/EVA, S-MgAl/APP/CFA/EVA, S-LaMgAl/APP/CFA/EVA, S-CeMgAl/APP/CFA/EVA, and S-NdMgAl/APP/CFA/EVA, respectively. All composites were treated in an oven (Beijing Xianghu Science and Development Company, Beijing, China) at 60 °C for 4 h before testing to remove adsorbed water from the surface.

### 2.3. Characterizations

X-ray diffraction (XRD) was carried out on a D/MAX 2200VPC diffractometer (Rigaku, Tokyo, Japan) with Cu Kα. Data were collected at the rate of 4°/min and step 0.02° at 40 kV and 30 mA. Fourier transform infrared (FTIR) spectra of LDH were collected using a Nicolet FTIR spectrometer (Thermo Nicolet, Madison, WI, USA) (KBr pellet method, 4 cm^−1^ resolution, number of scans was 32). The samples were scanned in the 2θ range of 10° to 70°. Scanning electron microscope (SEM) observations were carried out on an FEI-Sirion (FEI, Eindhoven, Holland) with a field emission of 20 kV. The transmission electron microscopy (TEM) was carried out with a HITACHI 1H-7650 (Hitachi, Tokyo, Japan) operating at the accelerating voltage of 100 kV. Thermal analysis was carried out with a thermogravimetric analyzer (TGA pyris 1, PerkinElmer Co, Ltd., Waltham, MA, USA) using a constant heating rate of 10 °C/min under a pure nitrogen atmosphere from 50–700 °C. The weight of the samples was kept within 3 and 4 mg. Vertical burning ratings (UL-94) were measured on an instrument (CZF-2, Jiangning Analysis Instrument Factory, Jiangning, China) with sample dimensions of 125 × 12.5 × 1.6 mm^3^ according to ISO 1210-1992. All cone calorimeter tests were carried out in a cone calorimeter (FTT0007, Hongkong Universal Technology Co, Hongkong, China) at an incident heat flux of 50 kW/m^2^ according to the ISO 5660-1 standard. The polymer sample (100 × 100 × 5 mm^3^) was placed horizontally on a balance holder. Before testing, aluminum foil was used to prevent ignition of the sides for the samples. The tensile strength and elongation at break of composites were evaluated according to the national standard GB/T 16421-1996 by using a material test machine (RGD-20A, Shenzhen Regear Instrument Cooperation, Shenzhen, China). A 1 mm thick sample was placed in a special mold to make a dumbbell-shaped standard sample before testing the tensile strength and elongation at break.

## 3. Results and Discussion

### 3.1. XRD and Morphology Analysis of the Modified LDHs

**Figure 3:**
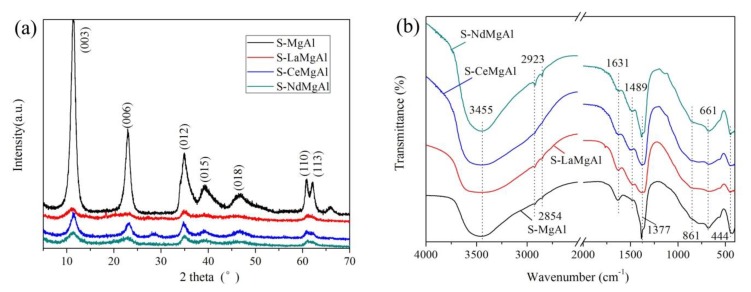
X-ray diffraction (XRD) and Fourier transform infrared (FTIR) results of the modified layered double hydroxides (LDHs): (**a**) XRD pattern; (**b**) FTIR spectra.

Figure 3a depicts the XRD patterns of the stearate surface-modified LDHs. Four LDHs revealed the typical layered double hydroxide structures with sharp and symmetrical characteristic diffraction peaks, namely (003), (006), (012), (015), (018), (110), and (113), which were indexed in a hexagonal lattice with an R3m rhombohedral space group symmetry. However, compared with S-MgAl, the diffraction peak resolutions of (110) and (113) of the other three LDHs were not high; this was attributed to the addition of REEs that reduced the orderly arrangement of anions within the brucite-type layers [22]. The basal spacings (d_basal_) of S-MgAl, S-LaMgAl, S-CeMgAl, and S-NdMgAl were 7.65 Å, 7.84 Å, 7.66 Å, and 7.84 Å calculated from the (003) diffraction, respectively. These values indicated that the interlayer anions were CO_3_^2−^ [23] besides OH^−^, without the coexistence of NO_3_^−^ or C_18_H_35_O^2−^ [24].

Figure 3b presents the FTIR spectra of the stearate surface-modified LDHs. All samples showed the band at 3455 cm^−1^, which was attributed to a large amount of OH^−^ between LDH layers. The band at 1631 cm^−1^ illustrated the bending vibration of –OH from crystal water. The appearance of this band was due to the electrostatic action and the hydrogen-bonding action between –OH on the layer board of the LDH and the anion or water molecule. The absorption peak at 1377 cm^−1^ was assigned to the stretching vibrations of C–O in CO_3_^2−^. Two bands at 661 cm^−1^ and 861 cm^−1^ were the in-plane bending vibration of C–O in CO_3_^2−^. The appearance of the band at 444 cm^−1^ was attributed to the lattice vibration of the metal –O. The band at 1489 cm^−1^ was due to the bridging–two–tooth complexation of CO_3_^2−^–CO_3_^2−^. The bands at 2923 cm^−^^1^ and 2854 cm^−^^1^ belonged to –CH_3_ and –CH– groups in sodium stearate [25]. According to the results of XRD and FTIR, the interlayer anions of synthetic samples were CO_3_^2−^ and sodium stearate absorbed on the surface of the LDH.

### 3.2. Morphology Analysis of the Modified LDHs

**Figure 4:**
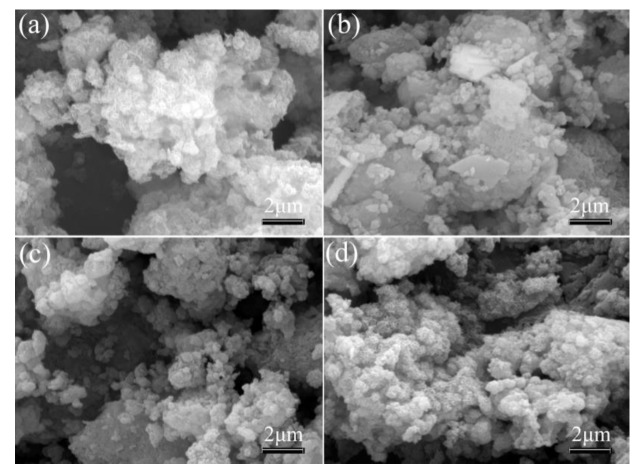
Scanning electron microscopy (SEM) images of the modified LDHs: (**a**) S-MgAl; (**b**) S-LaMgAl; (**c**) S-CeMgAl; (**d**) S-NdMgAl.

Figure 4 presents the SEM images of the four modified LDHs. The surfaces of all LDHs were uniformly coated by the stearate. The morphology results also confirmed that the stearate was uptaken on the surface of the LDHs, which was consistent with XRD and FTIR analysis.

**Figure 5:**
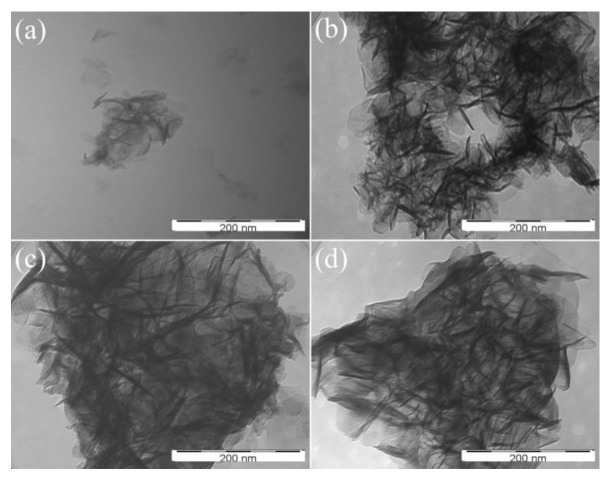
TEM images of the modified LDHs: (**a**) S-MgAl; (**b**) S-LaMgAl; (**c**) S-CeMgAl; (**d**) S-NdMgAl.

Figure 5 shows the TEM images of the four modified LDHs. All LDH samples showed the flake particle morphology. Furthermore, the four modified LDHs possessed the uniform and nanoscale crystal sizes. Moreover, their TEM images appeared aggregated, which was attributed to the effect of stearate on the surface of the LDH.

### 3.3. Thermal Analysis of Pure EVA and Its Composites

**Figure 6:**
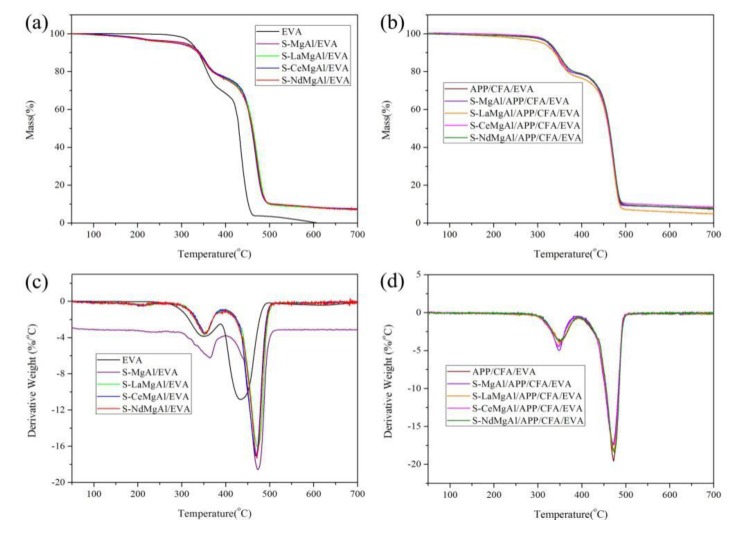
Thermogravimetric analysis (TGA) and differential thermogravimetry (DTG) curves of pure ethylene vinyl acetate (EVA) and its composites: (**a**,**b**) TGA curves; (**c**,**d**) DTG curves.

The thermogravimetric analysis (TGA) and differential thermogravimetry (DTG) curves of pure EVA and its composites are shown in Figure 6. From Figure 6a, it can be seen that pure EVA underwent two stages of decomposition, whereas all composites underwent three stages of decomposition. This could be attributed to the removal of the interlayer water molecules, dehydroxylation, and decomposition of the interlayer anions at 120–309 °C [26,27]. The initial decomposition temperature at 5 wt % weight loss (T_5%_) and final masses of char residues (F_m_) for pure EVA, S-MgAl/EVA, S-LaMgAl/EVA, S-CeMgAl/EVA, S-NdMgAl/EVA, APP/CFA/EVA, S-MgAl/APP/CFA/EVA, S-LaMgAl/APP/CFA/EVA, S-CeMgAl/APP/CFA/EVA, and S-NdMgAl/APP/CFA/EVA are shown in Table 2. The incorporation of a flame retardant changed the thermal degradation behavior to allow composites to have a lower T_5%_ in comparison with pure EVA; meanwhile, this promoted the formation of more char residue, and the F_m_ for composites was thus higher than that of pure EVA. However, the T_5%_ of S-LaMgAl/APP/CFA/EVA, S-CeMgAl/APP/CFA/EVA, and S-NdMgAl/APP/CFA/EVA was significantly higher than that of S-MgAl/EVA, S-LaMgAl/EVA, S-CeMgAl/EVA, S-NdMgAl/EVA, APP/CFA/EVA, and S-MgAl/APP/CFA/EVA. This fact indicates that the combination of APP, CFA, and LDH containing REEs can allow composites to achieve better thermal stability. From the DTG curves (Figure 6c,d), the maximum thermal degradation temperature of pure EVA was 437 °C, and the temperature at maximum weight loss rate (T_peak_) of all composites increased by more than 37 °C compared with that of pure EVA. This result confirmed that composites can achieve better thermal stability with the addition of flame retardant.

### 3.4. Flame Retardancy of EVA and Its Composites

#### 3.4.1. Vertical Burning (UL-94) and Cone Calorimetry Tests of Pure EVA and Its Composites

From Table 1, pure EVA, S-MgAl/EVA, S-LaMgAl/EVA, S-CeMgAl/EVA, and S-NdMgAl/EVA produced many drippings with flame during combustion. Therefore, these composites having a thickness of 1.6 mm failed to pass the UL-94 tests. In contrast, S-MgAl/APP/CFA/EVA, S-LaMgAl/APP/CFA/EVA, S-CeMgAl/APP/CFA/EVA, and S-NdMgAl/APP/CFA/EVA successfully passed UL-94 V-0 rating and produced no dripping. This could be attributed to the common flame-retardant effect between APP, CFA, and LDH containing REEs.

**Figure 7:**
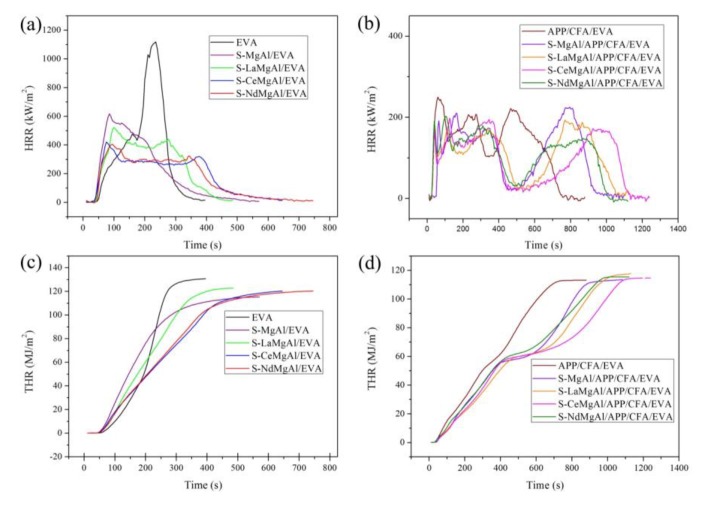
Heat release rate (HRR) and total heat release (THR) curves of pure EVA and its composites: (**a**,**b**) HRR curves; (**c**,**d**) THR curves.

**Figure 8:**
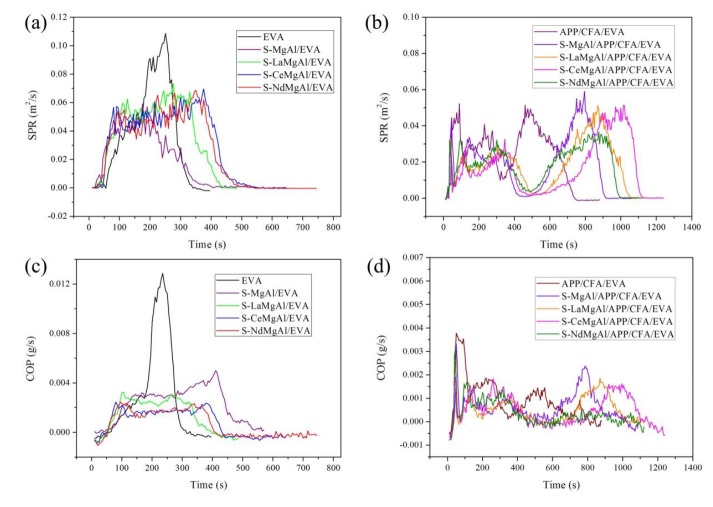
Smoke production rate (SPR) and production rate of CO (COP) curves of pure EVA and its composites: (**a**,**b**) SPR curves; (**c**,**d**) COP curves.

Cone calorimetry tests can simulate a real fire scenario [28]. Therefore, they were used to evaluate the materials’ flame retardancy. The curves of the heat release rate (HRR), total heat release (THR), smoke production rate (SPR), and production rate of CO (COP) are shown in Figure 7 and Figure 8. Before testing, aluminum foil was used to prevent ignition of the sides of the sample. The key data are listed in Table 2.

It is evident that the time to ignition (TTI) of composites was reduced compared with that of pure EVA. This was attributed to the decomposition of EVA, which led to an earlier evolution of volatiles, mainly acetic acid. The peak value of HRR (pk-HRR) of pure EVA was 1119.7 KW/m^2^. Compared with pure EVA, when incorporating LDH into the EVA matrix, the pk-HRR of S-MgAl/EVA, S-LaMgAl/EVA, S-CeMgAl/EVA, and S-NdMgAl/EVA was reduced to 617.9, 523.0, 521.4, and 404.8 KW/m^2^, and decreased by 44.8%, 53.3%, 53.4%, and 63.8%, respectively. All LDH containing REEs obviously reduced the fire strength in comparison with S-MgAl. The pk-HRR of APP/CFA/EVA and S-MgAl/APP/CFA/EVA was 249.0 KW/m^2^ and 225.0 KW/m^2^, compared with that of pure EVA, and the pk-HRR decreased by 77.8% and 79.9%, respectively. However, it is worth noting that the pk-HRR of S-LaMgAl/APP/CFA/EVA, S-CeMgAl/APP/CFA/EVA, and S-NdMgAl/APP/CFA/EVA decreased to 193.7, 198.2, and 202.8 KW/m^2^, respectively, and were all reduced by more than 82%. Moreover, the THR curves of all composites showed a decrease compared with that of pure EVA. The THR results demonstrated that the composite flame retardants reduced the spread of heat [29].

In a fire, the deadliest factor is not the heat but the smoke and toxic gases produced, such as CO [30]. The peak values of SPR (pk-SPR) and peak values of COP (pk-COP) for pure EVA, S-MgAl/EVA, S-LaMgAl/EVA, S-CeMgAl/EVA, S-NdMgAl/EVA, APP/CFA/EVA, S-MgAl/APP/CFA/EVA, S-LaMgAl/APP/CFA/EVA, S-CeMgAl/APP/CFA/EVA, and S-NdMgAl/APP/CFA/EVA are listed in Table 2. It can be seen that S-LaMgAl/APP/CFA/EVA, S-CeMgAl/APP/CFA/EVA, and S-NdMgAl/APP/CFA/EVA showed lower values of pk-HRR, THR, pk-SPR, and pk-COP. Furthermore, the pk-HRR, pk-SPR and pk-COP of S-LaMgAl/APP/CFA/EVA, S-CeMgAl/APP/CFA/EVA, and S-NdMgAl/APP/CFA/EVA were lower than those reported for APP/CFA/LDH/EVA without REEs [13]. The results showed that the addition of REEs allowed composites to achieve a better flame retardancy, smoke release, and CO suppression. The char residues of composites were higher than those of pure EVA, which all increased more than three times. The increases in char residues were one of the main reasons of the improvement noted for HRR, THR, SPR, and COP.

The average mass loss rate (AMLR) values of pure EVA and its composites are shown in Table 2. The AMLR values decreased by 43%, 33%, 53%, 50%, 71%, 73%, 80%, 82%, and 78% compared with that of pure EVA, respectively. The results of AMLR showed the condensed-phase fire retardant action of APP, CFA, and LDH containing REEs in the composites.

#### 3.4.2. Digital Photographs of Residues after Cone Calorimeter Tests

**Figure 9:**
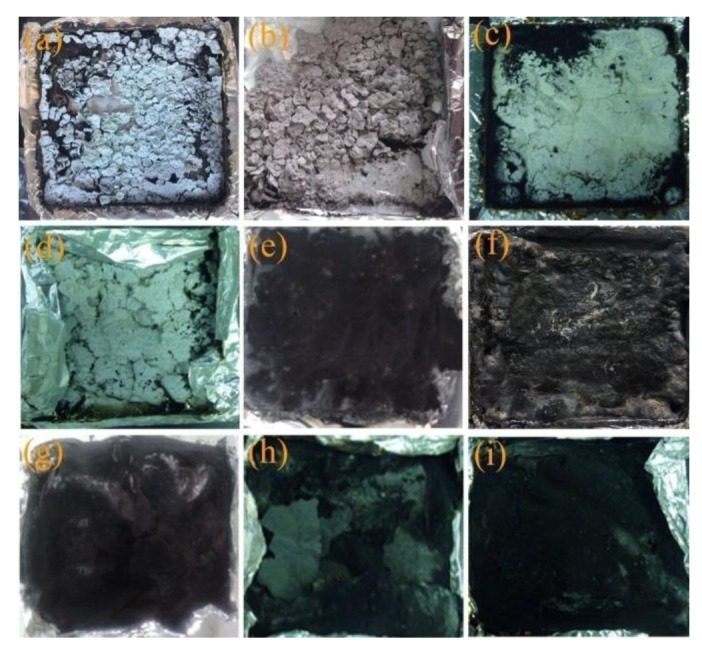
Digital photographs of char residues after cone calorimeter tests for the composites:(**a**) S-MgAl/EVA; (**b**) S-LaMgAl/EVA; (**c**) S-CeMgAl/EVA; (**d**) S-NdMgAl/EVA; (**e**) APP/CFA/EVA; (**f**) S-MgAl/APP/CFA/EVA; (**g**) S-LaMgAl/APP/CFA/EVA; (**h**) S-CeMgAl/APP/CFA/EVA; (**i**) S-NdMgAl/APP/CFA/EVA.

The char layer was considered an important influence on flame retardancy. In order to know the level of flame retardancy, the digital photographs of residues after the cone calorimeter tests are shown in Figure 9. Pure EVA had almost no residue remaining. The char layers of S-MgAl/EVA, S-LaMgAl/EVA, S-CeMgAl/EVA, and S-NdMgAl/EVA were fragmentary and not continuous. The char layer of APP/CFA/EVA was continuous, but the char layer was loose and had some small holes on the surface. Finally, the char layer of S-MgAl/APP/CFA/EVA was not continuous. Relatively speaking, the char layers of S-LaMgAl/APP/CFA/EVA, S-CeMgAl/APP/CFA/EVA, and S-NdMgAl/APP/CFAEVA were continuous and compact. The more compact and uniform char layers could reduce the volatile release and better limit the heat and gas transfer [31]. Hence, S-LaMgAl/APP/CFA/EVA, S-CeMgAl/APP/CFA/EVA, and S-NdMgAl/APP/CFA/EVA achieved a better flame retardancy.

#### 3.4.3. Morphological Analysis of Cone Calorimetric Residues

**Figure 10:**
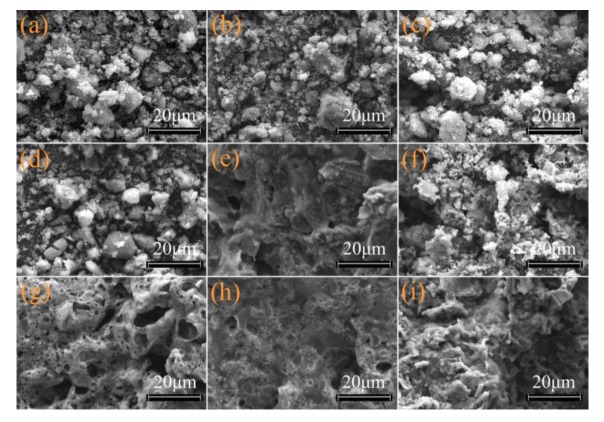
SEM images of the cone calorimetric residues: (**a**) S-MgAl/EVA; (**b**) S-LaMgAl/EVA; (**c**) S-CeMgAl/EVA; (**d**) S-NdMgAl/EVA; (**e**)APP/CFA/EVA; (**f**) S-MgAl/APP/CFA/EVA; (**g**) S-LaMgAl/APP/CFA/EVA; (**h**) S-CeMgAl/APP/CFA/EVA; (**i**) S-NdMgAl/APP/CFA/EVA.

The SEM images of the cone calorimetric residues are shown in Figure 10. The residues of S-MgAl/EVA, S-LaMgAl/EVA, S-CeMgAl/EVA, and S-NdMgAl/EVA were mainly metal oxides, which resulted in no dense carbon layers. In contrast, APP/CFA/EVA, S-MgAl/APP/CFA/EVA, S-LaMgAl/APP/CFA/EVA, S-CeMgAl/APP/CFA/EVA, and S-NdMgAl/APP/CFA/EVA had dense and continuous carbon layers. Compared with APP/CFA/EVA and S-MgAl/APP/CFA/EVA, many bubbles of S-LaMgAl/APP/CFA/EVA, S-CeMgAl/APP/CFA/EVA, and S-NdMgAl/APP/CFA/EVA residues were observed, as shown in Figure 10g–i. This result indicated that the incorporation of LDH containing REEs led to the release of more flame-retardant gases. The continuous and compact layers of S-LaMgAl/APP/CFA/EVA, S-CeMgAl/APP/CFA/EVA, and S-NdMgAl/APP/CFA/EVA were the main reason for the flame retardancy of the condensed phase.

### 3.5. Mechanical Property Analysis of Pure EVA and Its Composites

**Figure 11:**
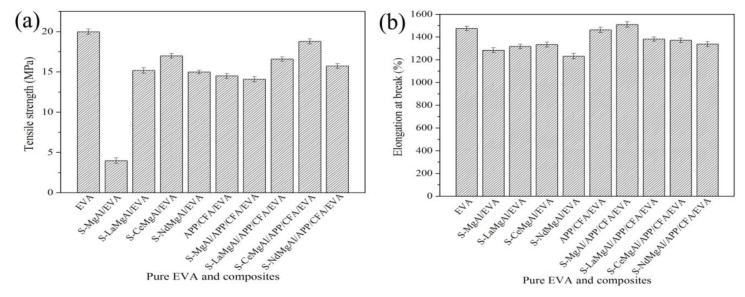
Mechanical properties of pure EVA and its composites: (**a**) tensile strength; (**b**) elongation at break.

Before testing the tensile strength and elongation at break for all samples, a 1 mm thick sample was placed in a special mold to make a dumbbell-shaped standard sample. The tests were conducted at the speed of 50 mm/min and gauge was 25 mm. All data points regarding both the tensile strength and the elongation at break were the mean values of 10 test data. Further, the 10 test data were within the error of the measurements. The tensile strength and elongation at break of pure EVA and its composites are presented in Figure 11.

As is well known, the incorporation of a flame retardant can result in changes to the mechanical properties for a polymer matrix (as shown in Figure 11). Due to the addition of 20% flame retardants, compared with pure EVA, the tensile strength values of S-MgAl/EVA, S-LaMgAl/EVA, S-CeMgAl/EVA, S-NdMgAl/EVA, APP/CFA/EVA, S-MgAl/APP/CFA/EVA, S-LaMgAl/APP/CFA/EVA, S-CeMgAl/APP/CFA/EVA, and S-NdMgAl/APP/CFA/EVA decreased significantly. However, it should be pointed out that, compared with S-MgAl/EVA, S-LaMgAl/EVA, S-CeMgAl/EVA, S-NdMgAl/EVA, APP/CFA/EVA, and S-MgAl/APP/CFA/EVA, composites containing REEs (S-LaMgAl/APP/CFA/EVA, S-CeMgAl/APP/CFA/EVA, and S-NdMgAl/APP/CFA/EVA) still retained relatively good strength. As Figure 11b shows, S-MgAl/APP/CFA/EVA obtained the highest ductility. With the incorporation of REEs, the elongation at break of S-LaMgAl/APP/CFA/EVA, S-CeMgAl/APP/CFA/EVA, and S-NdMgAl/APP/CFA/EVA decreased. Thus, REEs reduced the compatibility between APP, CFA, and LDH. However, the elongation at break of S-LaMgAl/APP/CFA/EVA, S-CeMgAl/APP/CFA/EVA, and S-NdMgAl/APP/CFA/EVA was higher than that of S-MgAl/EVA, S-LaMgAl/EVA, S-CeMgAl/EVA, and S-NdMgAl/EVA. This confirmed that the combination between APP, CFA, and LDH containing REEs not only allowed composites to maintain favorable mechanical properties but also to retain the required flame retardancy and smoke suppression.

## 4. Conclusions

The combination of XRD, FTIR, and SEM results indicated that stearate was grafted onto the surfaces of the LDHs. Compared with EVA, the pk-HRR, THR, pk-SPR, and pk-COP values for S-LaMgAl/APP/CFA/EVA, S-CeMgAl/APP/CFA/EVA, and S-NdMgAl/APP/CFA/EVA all decreased, especially the pk-HRR that decreased by more than 82%. This was attributed to the combination of APP, CFA, and LDH containing REEs which formed compact char layers and released more flame-retardant gases to improve the flame retardancy and smoke suppression of the EVA matrix. From the SEM images of residues, the values of AMLR indicated the condensed-phase fire retardant action in the composites. Meanwhile, after incorporating the composite flame retardant, the mechanical properties were still well maintained. Hence, the formed novel flame retardants are promising additive agents in terms of flame retardancy.

## Figures and Tables

**Table 1:** Ingredients of the composites.

Sample	LDH (%)	APP (%)	CFA (%)	EVA (%)	UL-94 Rating (at 1.6 mm)	Phenomenon
EVA	-	-	-	100	Fail	Dripping
S-MgAl/EVA	20	-	-	80	Fail	Dripping
S-LaMgAl/EVA	20	-	-	80	Fail	Dripping
S-CeMgAl/EVA	20	-	-	80	Fail	Dripping
S-NdMgAl/EVA	20	-	-	80	Fail	Dripping
APP/CFA/EVA	-	15	5	80	Fail	Dripping
S-MgAl/APP/CFA/EVA	4	12	4	80	V-0	No Dripping
S-LaMgAl/APP/CFA/EVA	4	12	4	80	V-0	No Dripping
S-CeMgAl/APP/CFA/EVA	4	12	4	80	V-0	No Dripping
S-NdMgAl/APP/CFA/EVA	4	12	4	80	V-0	No Dripping

**Table 2:** TGA, DTG, and cone calorimetric data.

Samples	T_5%_ (°C)	F_m_ (wt %)	TTI (s)	pk-HRR (KW/m^2^)	THR (MJ/m^2^)	pk-SPR (m^2^/s)	pk-COP (g/s)	AMLR (g/s)	Residue (wt %)
EVA	331.0	0	48	1119.7 ± 8.2	130.8 ± 6.5	0.109 ± 0.007	0.013 ± 0.001	20.6	3.2 ± 0.5
S-MgAl/EVA	309.5	7.6	43	617.9 ± 7.4	115.4 ± 5.7	0.059 ± 0.002	0.005 ± 0.001	11.7	13.6 ± 1.1
S-LaMgAl/EVA	289.5	6.9	41	523.0 ± 7.6	122.8 ± 4.9	0.069 ± 0.005	0.003 ± 0.001	13.8	11.6 ± 1.4
S-CeMgAl/EVA	288.4	7.5	36	521.4 ± 6.7	120.3 ± 5.2	0.070 ± 0.009	0.003 ± 0.001	9.7	10.7 ± 0.9
S-NdMgAl/EVA	294.3	7.1	44	404.8 ± 7.2	120.4 ± 5.4	0.069 ± 0.006	0.003 ± 0.001	10.3	12.8 ± 0.7
APP/CFA/EVA	324.2	7.4	26	249.0 ± 5.6	113.1 ± 4.9	0.052 ± 0.007	0.002 ± 0.001	5.9	12.2 ± 0.6
S-MgAl/APP/CFA/EVA	312.7	7.8	31	225.0 ± 5.4	113.4 ± 4.7	0.059 ± 0.005	0.003 ± 0.001	5.5	12.5 ± 0.9
S-LaMgAl/APP/CFA/EVA	327.3	4.9	27	193.7 ± 6.2	117.7 ± 3.8	0.051 ± 0.002	0.001 ± 0.001	4.2	13.1 ± 0.8
S-CeMgAl/APP/CFA/EVA	327.1	8.4	26	198.2 ± 5.6	114.7 ± 3.2	0.052 ± 0.006	0.002 ± 0.001	3.7	11.8 ± 0.5
S-NdMgAl/APP/CFA/EVA	326.0	7.8	25	202.8 ± 7.3	115.4 ± 2.9	0.037 ± 0.008	0.002 ± 0.001	4.4	11.6 ± 0.4

## References

[1] Liu J.J., Zhou K.Q., Tang G., Wang B.B., Gui Z., Yuen R.K.K., Hu Y. (2018). Synthesis of Co_3_(HPO_4_)_2_(OH)_2_ nanosheets and its synergistic effect with intumescent flame retardants in ethylene-vinyl acetate copolymer. Polym. Compos..

[2] Kalali E.N., Juan S.D., Wang X., Nie S.B., Wang R., Wang D.Y. (2015). Comparative study on synergistic effect of LDH and zirconium phosphate with aluminum trihydroxide on flame retardancy of EVA composites. J. Therm. Anal. Calorim..

[3] Qi J.J., Wen Q.Z., Zhu J.H. (2019). Synergistic effect of intumescent flame retardant system consisting of hexophenoxy cyclotriphosphazene and ammonium polyphosphate on methyl ethyl silicone rubber. Mater. Lett..

[4] Lee J.H., Zhang W., Ryu H.J., Choi G., Choi J.Y., Choy J.H. (2019). Enhanced thermal stability and mechanical property of EVA nanocomposites upon addition of organo-intercalated LDH nanoparticles. Polymer.

[5] Smalenskaite A., Kaba M.M., Grigoraviciute-Puroniene I., Mikoliunaite L., Zarkov A., Ramanauskas R., Morkan I.A., Kareiva A. (2019). Sol–Gel Synthesis and Characterization of Coatings of Mg-Al Layered Double Hydroxides. Materials.

[6] Li C.F., Zhao G.Q., Zhang T.H., Yan T., Zhang C.Y., Wang L.J., Liu L.K., Zhou S., Jiao F.P. (2020). A novel Ag@TiON/CoAl-layered double hydroxide photocatalyst with enhanced catalytic memory activity for removal of organic pollutants and Cr (VI). Appl. Surf. Sci..

[7] Ma R.Y., Tang P.G., Feng Y.J., Li D.Q. (2019). UV absorber co-intercalated layered double hydroxides as efficient hybrid UV-shielding materials for polypropylene. Dalton Trans..

[8] Qian Y., Zhou S.J., Chen X.L. (2017). Flammability and thermal degradation behavior of ethylene-vinyl acetate/layered double hydroxides/zinc borate composites. Polym. Adv. Technol..

[9] Ma H.Y., Tong L.F., Xu Z.B., Fang Z.P. (2008). Intumescent flame retardant-montmorillonite synergism in ABS nanocomposites. Appl. Clay Sci..

[10] Abdelkhalik A., Makhlouf G., Hassan M.A. (2019). Manufacturing, thermal stability, and flammability properties of polypropylene containing new single molecule intumescent flame retardant. Polym. Adv. Technol..

[11] Yang R., Ma B.B., Zhang X., Li J.C. (2019). Fire retardance and smoke suppression of polypropylene with a macromolecular intumescent flame retardant containing caged bicyclic phosphate and piperazine. J. Appl. Polym. Sci..

[12] Li B., Jia H., Guan L.M., Bing B.C., Dai J.F. (2009). A novel intumescent flame-retardant system for flame-retarded LLDPE/EVA composites. J. Appl. Polym. Sci..

[13] Wang L.L., Xu M.J., Shi B.L., Li B. (2016). Flame retardance and smoke suppression of CFA/APP/LDHs/EVA composite. Appl. Sci..

[14] Zhang J., Zhao N., Wei W., Sun Y.H. (2010). Partial oxidation of methane over Ni/Mg/Al/La mixed oxides prepared from layered double hydrotalcites. Int. J. Hydrogen Energy.

[15] Pan J.H., Zhou C.C., Tang M.C., Cao S.S., Liu C., Zhang N.N., Wen M.Z., Luo Y.L., Hu T.T., Ji W.S. (2019). Study on the modes of occurrence of rare earth elements in coal fly ash by statistics and a sequential chemical extraction procedure. Fuel.

[16] Borai E.H., Hamed M.G., El-kamash A.M., Siyam T., El-Sayed G.O. (2015). Template polymerization synthesis of hydrogel and silica composite for sorption of some rare earth elements. J. Colloid Interface Sci..

[17] Guo K., Man Z.Y., Cao Q.G., Chen H.H., Guo X.X., Zhao J.T. (2011). Effects of rare-earth substitution on the stability and electronic structure of REZnOSb (RE = La–Nd, Sm–Gd) investigated via first-principles calculations. Chem. Phys..

[18] Babizhetskyy V., Simon A., Mattausch H., Hiebl K., Zheng C. (2010). New ternary rare-earth metal boride carbides R15B4C14 (R = Y, Gd–Lu) containing BC2 units: Crystal and electronic structures, magnetic properties. J. Solid State Chem..

[19] Li B., Xu M.J. (2006). Effect of a novel charring–foaming agent on flame retardancy and thermal degradation of intumescent flame retardant polypropylene. Polym. Degrad. Stabil..

[20] Li B., Xu M.J., Zhang X.C. (2006). Macromolecular Triazine Carbon-Forming Agent and Synthesis Method.

[21] Dai J.F., Li B. (2010). Synthesis, thermal degradation, and flame retardance of novel triazine ring-containing macromolecules for intumescent flame retardant polypropylene. J. Appl. Polym. Sci..

[22] Benito P., Labajos F.M., Rives V. (2006). Microwave-treated layered double hydroxides containing Ni^2+^ and Al^3+^: The effect of added Zn^2+^. J. Solid State Chem..

[23] He H.M., Kang H.L., Ma S.L., Bai Y.X., Yang X.J. (2010). High adsorption selectivity of ZnAl layered double hydroxides and the calcined materials toward phosphate. J. Colloid Interface Sci..

[24] Kloprogge J.T., Wharton D., Hickey L., Frost R.L. (2002). Infrared and Raman study of interlayer anions CO_3_^2−^, NO_3_^−^, SO_4_^2−^ and ClO_4_^−^ in Mg/Al-hydrotalcite. Am. Mineral..

[25] Zheng Z.H., Liu Y.H., Dai B.Y., Meng C.Y., Guo Z.X. (2019). Carbon dots: Synthesis, formation mechanism, fluorescence origin and sensing applications. Carbohydr. Polym..

[26] Özgümüş S., Gök M.K., Bal A., Güçlü G. (2013). Study on novel exfoliated polyampholyte nanocomposite hydrogels based on acrylic monomers and Mg–Al–Cl layered double hydroxide: Synthesis and characterization. Chem. Eng. J..

[27] Wang L.L., Zhang M.L., Li B. (2016). Thermal analysis and flame-Retarded mechanism of composites composed of ethylene vinyl acetate and layered double hydroxides containing transition metals (Mn, Co, Cu, Zn). Appl. Sci..

[28] Xu W.Z., Wang X.L., Wu Y., Li W., Chen C.Y. (2019). Functionalized graphene with Co-ZIF adsorbed borate ions as an effective flame retardant and smoke suppression agent for epoxy resin. J. Hazard. Mater..

[29] Kong Q.H., Wu T., Zhang H.K., Zhang Y., Zhang M.M., Si T.Y., Yang L., Zhang J.H. (2017). Improving flame retardancy of IFR/PP composites through the synergistic effect of organic montmorillonite intercalation cobalt hydroxides modified by acidified chitosan. Appl. Clay Sci..

[30] Cai T.M., Wang J.L., Zhang C.H., Cao M., Jiang S.J., Wang X., Wang B.B., Hu W.Z., Hu Y. (2020). Halogen and halogen-free flame retarded biologically-based polyamide with markedly suppressed smoke and toxic gases releases. Compos. Part B-Eng..

[31] Song K.L., Ganguly I., Eastin I., Dichiara A. (2020). High temperature and fire behavior of hydrothermally modified wood impregnated with carbon nanomaterials. J. Hazard. Mater..

